# Mesoporous nanoplatform integrating photothermal effect and enhanced drug delivery to treat breast cancer bone metastasis

**DOI:** 10.3389/fchem.2022.1088823

**Published:** 2022-11-30

**Authors:** Wujun Miao, Yunfan Ti, Jingwei Lu, Jianning Zhao, Bin Xu, Liang Chen, Nirong Bao

**Affiliations:** ^1^ Department of Orthopedics, Affiliated Jinling Hospital, Medical School of Nanjing University, Nanjing, China; ^2^ Materdicine Lab, School of Life Sciences, Shanghai University, Shanghai, China

**Keywords:** breast cancer, bone metastasis, photothermal therapy, drug delivery, mesoporous nanoplatform

## Abstract

Bone metastatic breast cancer has severely threatened the survival and life quality of patients. Due to the suboptimal efficacy of anti-metastatic chemotherapeutic drugs and the complicated bone marrow microenvironments, effective treatment of metastatic breast cancer remains challenging for traditional clinical approaches. In this work, we developed a mesoporous nanoplatform (m-CuS-PEG) with the co-loading of CuS nanodots and a chemotherapeutic drug cisplatin for the combined photothermal-chemotherapy of bone-metastasized breast cancer. The CuS nanodots were decorated onto mesoporous silica (m-SiO_2_) surface with dendritic mesoporous channels, into which the cisplatin was accommodated. The carboxyl-terminated poly (ethylene glycol) (PEG) was further functionalized onto the surface to obtain the functional nanoplatform m-CuS-PEG. The drug release of the loaded cisplatin exhibited pH- and thermal-dual responsive manner. The attached CuS nanodots rendered the mesoporous nanoplatform with high photothermal conversion ability. Upon irradiation with a near-infrared laser in the second near-infrared (NIR-II) window, m-CuS-PEG dispersions exhibited rapid temperature elevation and high photostability. The results revealed that m-CuS-PEG had excellent biocompatibility. The cisplatin-loaded m-CuS-PEG not only showed superior cancer cell-killing effects, but also significantly inhibit the growth of metastatic tumors. The tumor-induced bone destruction was also dramatically attenuated by the mesoporous nanoplatform-mediated combined therapy. Overall, the developed functional nanoplatform integrates photothermal therapy and efficient chemotherapeutic drug delivery to offer an alternative approach for combating breast cancer bone metastasis.

## Introduction

Breast cancer is one of the leading causes of cancer deaths in women worldwide. Patients with advanced breast cancer often inevitably suffer from bone metastases. (Cicek and Oursler 2006; Coleman 2012; Ross et al., 2017) Bone metastases may result in a series of life-threatening complications, such as pathological fracture, neurological compression, and hypercalcaemia. (Suva et al., 2011; Suvannasankha and Chirgwin 2014; Tahara et al., 2019) Conventional surgery resection can eliminate the primary tumor lesions but fails to prevent the secondary metastases and high-risk recurrence. (Adjei et al., 2018; Wang et al., 2020) Chemotherapy, the most commonly used treatment for bone metastases, has inherent limitations such as severe adverse effects and lower delivery efficacy. (Li et al., 2017) The complicated microenvironment of bone marrow also promotes the tumor cells’ survival and mediates the drug resistance of cancer cells to attenuate the therapeutic efficacy. (Croucher et al., 2016) Because of this, effective treatment of breast cancer bone metastases frequently needs high-dose chemotherapeutic drugs accompanied by high toxicity. Thus, it is urgent to develop therapeutic approaches that effectively and simultaneously eradicate bone metastatic tumors. (Huang et al., 2021).

Nanobiomaterials have emerged as an all-rounder to implement a wide range of therapy, including stimuli-responsive drug delivery, photodynamic therapy, photothermal therapy (PTT) and catalytic therapy. (Huo et al., 2017; Garbayo et al., 2020; Liu et al., 2020; Qin et al., 2021) Among them, PTT has attracted increasing attention in cancer therapy due to minimal invasiveness, ease to operation, low side effects, and high spatiotemporal selectivity. (Gao et al., 2021) PTT utilizes photothermal agents to convert near-infrared (NIR) photonic energy into heat energy for the local ablation of tumor tissues, offering a more effective and controllable approach to eradicate bone metastatic foci with reduced side effects. (Lin et al., 2015; Wang et al., 2015; Wang et al. 2017; Wang et al. 2018; Zhao et al., 2020; Ge et al., 2022) To realize more efficient PTT, the selected photothermal agents should possess both outstanding photothermal conversion efficiency and biocompatibility. Copper sulfide (CuS) nanoparticles have been extensively developed for PTT due to their attractive features, high photothermal conversion efficiency and photostability, strong plasmon resonance absorption, favorable biocompatibility, and the feasibility of metabolism excretion. (Yang et al., 2016; Lv et al., 2017; Feng et al., 2021) Owing to the plasmon resonance absorption, the high NIR absorption of CuS nanoparticles extends to the second NIR (NIR-II) biowindow, which can be leveraged for NIR-II PTT with deep tissue penetration. (An et al., 2021; Sun et al., 2021) The efficient deep-penetrating photothermal ablation is also beneficial for eradicating bone metastasized tumors. On the other hand, single-modality PTT may result in suboptimal efficacy for combating advanced breast cancer. (Li et al., 2017; Sun et al., 2019; Huang et al., 2020; Jiang et al., 2022) Collectively, rational design and development of functional nanoplatforms for combining photothermal and targeted delivery of chemotherapeutic drugs are highly desired for the efficient treatment of bone metastasized tumors.

**FIGURE 1:**
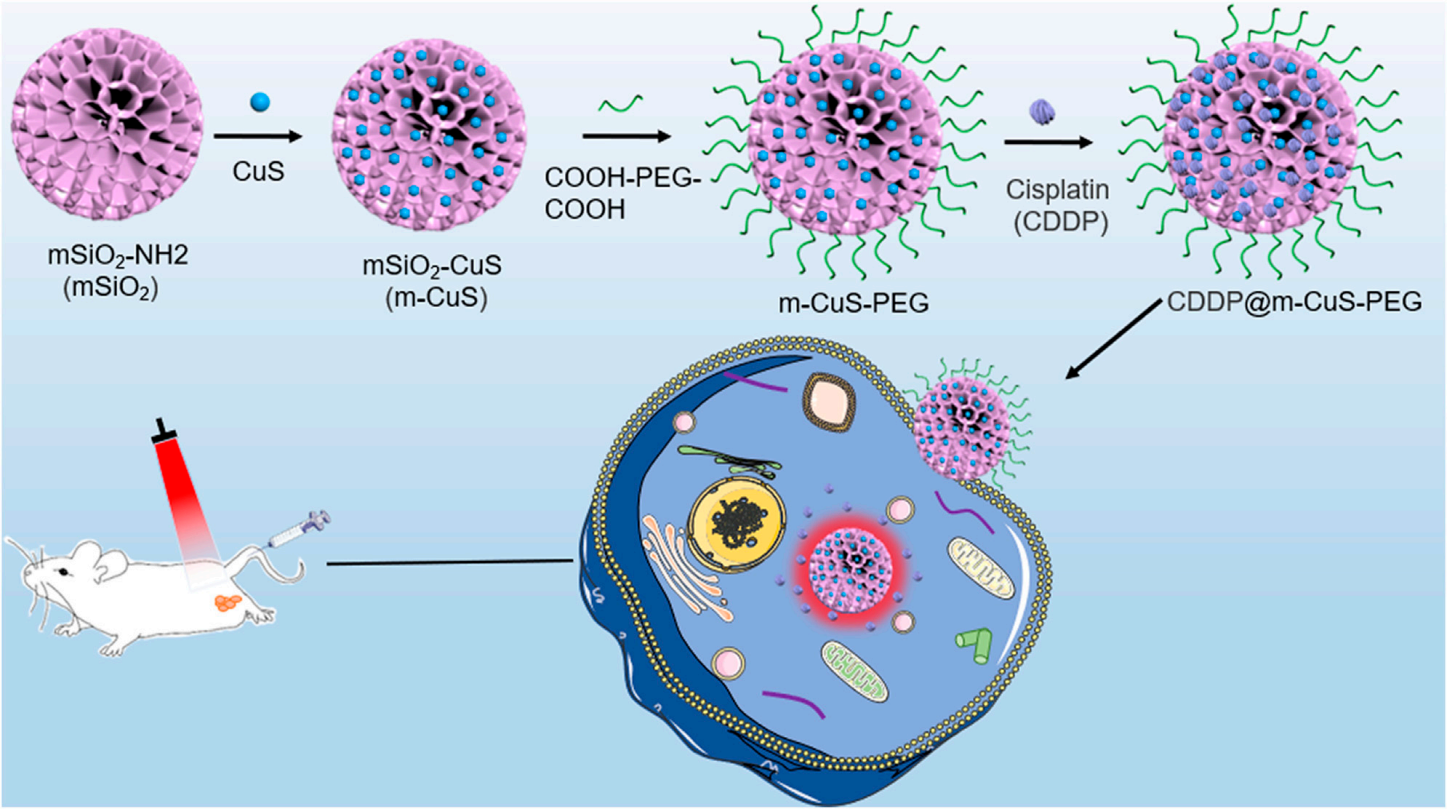
Schematic illustration of the synthesis process of CDDP@m-CuS-PEG NPs and the bone targeting delivery system, CDDP@m-CuS-PEG NPs.

Here, aiming at nanomedicine for high-performance metastasized tumor therapy, we fabricated a functional nanoplatform composed of mesoporous silica (m-SiO_2_) and ultrasmall CuS nanoparticles for the combined photothermal-chemotherapy of breast cancer bone metastases (Figure 1). Considering their high porosity and easy functionalization, the m-SiO_2_ nanoparticles with dendritic mesoporous channels were employed as the supercarrier for co-loading the inorganic photothermal agent and chemotherapeutic drug cisplatin (CDDP). In addition, the dicarboxylic poly (ethylene glycol) (COOH-PEG-COOH) was modified on the surface of the nanocomposites (CDDP@m-CuS-PEG). It is noted that the carboxylic groups-terminated surface can improve the bone affinity of the developed mesoporous nanoplatform. Taken together, the mesoporous nanoplatform rationally integrated the photothermal ability of CuS nanoparticles and the therapeutic efficacy of cisplatin. The remarkable combinational therapeutic effects and desirable biocompatibility of this functional nanoplatform make them highly promising for the efficient treatment of breast cancer bone metastases.

## Materials and methods

### Preparation of ultrasmall CuS nanodots

Ultrasmall CuS nanodots were prepared according to a previous study. (Xiao et al., 2013) Briefly, 2 ml of Na_2_S solution was added into 100 ml of CuCl_2_•2H_2_O aqueous solution containing 20 mg of sodium citrate. The mixture was vigorously stirred at room temperature for 5 min, followed by heating at 90°C for 10 min. The obtained CuS nanodots were directly stored at 4°C for further use.

### Preparation of m-SiO_2_ nanoparticles

The m-SiO_2_ nanoparticles with dendritic mesoporous channels were prepared by a bi-phase stratification approach. In a typical process, 1.5 g of cetyltrimethylammonium bromide (CTAB) and 0.1 ml of triethanolamine (TEA) were dissolved in 60.0 ml water and heated at 60°C. After that, a mixed solution of 8.5 ml of cyclohexane and 1.5 ml of tetraethoxysilane (TEOS) was added. The reaction was further proceeded overnight at 60°C. High-speed centrifugation was used to collect the prepared m-SiO_2_ nanoparticles. The produces were repeatedly refluxed in acidic ethanol solution to remove the surfactants. Last, the pure m-SiO_2_ nanoparticles were dispersed in 100 ml of ethanol and 0.2 ml of 3-aminopropyltriethoxysilane (APTES) was added. The mixture was stirred at 80°C overnight to obtain amine-functionalized m-SiO_2_.

### Preparation the mesoporous nanoplatform

Aminated m-SiO_2_ nanoparticles (10 mg) was dispersed in 10 ml of water, into which 20 ml of as-prepared CuS aqueous solution (∼5 mg/ml) was added and the mixture was further stirred at room temperature for 4 h. The CuS-decorated m-SiO_2_ (m-CuS) was isolated by high-speed centrifugation. To conjugate the carboxyl-terminated PEG onto m-CuS, 20 mg of COOH-PEG-COOH (Mw ∼2000) was dissolved in 5 ml of water, and 12 mg of EDC and 8.6 mg of NHS was added into the solution. The activation process maintained for 2 h. Subsequently, the m-CuS dispersed in water at a concentration of 10 mg/ml was added into the former solution. The mixture was reacted for 24 h to obtain m-CuS-PEG. Finally, the m-CuS-PEG was collected by centrifugation and washed with double-ionized (DI) water for several times. The purified m-CuS-PEG was stored at 4°C for further use.

Afterward, 20 mg of m-CuS-PEG was dispersed in 10 ml of water for cisplatin loading. Also, the drugs cisplatin was totally dissolved and aged in water to obtain aqueous solution of hydrolytic cisplatin (1 mg/ml). Then 4 ml of cisplatin solution was added into 10 ml of m-CuS dispersion. The mixture was stirred in dark for 24 h. The drug-loaded m-CuS-PEG was collected and washed by high-speed centrifugation. The loading content of cisplatin was measured by inductively coupled plasma optical emission spectrometry (ICP-OES).

### Drug release assessment of CDDP@m-CuS-PEG

To investigate the drug release behavior, three parallel samples of CDDP@m-CuS-PEG were dispersed in 2 ml of phosphate buffer solution (PBS) with different pH conditions (5.5 and 7.4), which were further sealed in dialysis bags (Cut-off Mw ∼3,500) and immersed in 10 ml PBS buffer with the same pH conditions. The samples were shake at 37°C and the solution was withdrawn at specific time points. Fresh PBS was added back to maintain the total volume unchanged. The resulting supernatant was finally collected for HPLC detection. In addition, to inspect the effect of NIR laser on drug release efficiency, the drug release experiments were carried out by incubating the CDDP@m-CuS-PEG at 37 and 42°C. At the indicated time points, the suspension was vertically irradiated with an 808 nm, 1 W/cm^2^ NIR laser for 10 min. Finally, the supernatants before and after laser irradiation were used for further detection.

### Evaluation of photothermal effect of m-CuS-PEG

The photothermal effect of m-CuS-PEG was assessed by an infrared thermal imaging. 0.2 ml of aqueous suspension of m-CuS-PEG were irradiated with an 808 nm laser at different power densities (0.5, 1.0, and 2 W/cm^2^), and the infrared thermal images were continuously recorded. The same protocols were also applied to evaluate the temperature change of different concentrations of m-CuS-PEG dispersions (0.2 and 0.4 mg/ml) under laser irradiation. The thermal stability of m-CuS-PEG was inspected by exposing the aqueous suspensions to five on-off cycles of laser irradiation-natural cooling.

### *In vitro* cytotoxicity of m-CuS-PEG

MDA-MB-231 breast cancer cells were seeded in 96-well plates at a density of 10^4^ cells per well. After 24 h, complete culture medium containing m-CuS-PEG at different concentrations (0, 1, 10, 20, 50, 100, 200, and 400 μg/ml) were added. The cells were incubated with the samples for 24 h or 72 h. The old medium was removed and the cells were slightly rinsed with PBS for two times. Then 10 μl CCK-8 solution (Dojindo, Japan) was added into each well for 2 h incubation, followed by determination of absorbance at 450 nm using a microplate reader (Bio-Rad, Hercules, CA, United States). Three parallel experiments were conducted for each group.

### *In vitro* blood compatibility assay

First, red blood cells were extracted from 6-week-old rats and centrifuged at 3,500 rpm for 10 min to remove supernatant. Then, PBS was added for washing and the concentration of red blood cells was adjusted to 16%. The m-CuS-PEG was diluted with PBS for 5, 10, 50, 100, 200, 400, 800 μg/ml solution. 100 μl red blood cell solution was added with 1 ml m-CuS-PEG solution of different concentrations, and pure water was added as positive control and PBS as negative control. After incubation for 8 h, absorbance was measured at 540 nm. The hemolysis rate was calculated by the following:

$$Hemolysis\left(\%\right)=\frac{A_{samples}-A_{negative}}{A_{postive}-A_{negative}}$$

Note that $A_{samples}$ was the absorbance of samples incubated with nanoparticles measured at different time intervals. $A_{postive}$ was the absorbance of samples incubated with pure water and $A_{negative}$ was the absorbance of samples incubated with PBS.

### *In vitro* therapeutic efficacy evaluation

MDA-MB-231 cells were seeded in 96-well plates at a density of 10^4^ cells per well and cultured overnight. After that, the cells were treated with m-CuS-PEG, CDDP@m-CuS-PEG, m-CuS-PEG plus NIR laser (808 nm, 1 W/cm^2^), CDDP@m-CuS-PEG plus NIR laser. The cells were incubated with the materials for 6 h and then subjected to the NIR laser irradiation. The m-CuS-PEG was equally 400 μg/ml for each group. After the corresponding treatments, the cells were further cultured for 18 h. Then the cell viability of each group was evaluated by the standard CCK-8 assay.

### *In vivo* biosafety of m-CuS-PEG

The biosafety of m-CuS-PEG was evaluated by intravenous injection of m-CuS-PEG (3 mg/ml, 200 μl per mouse) in healthy C57BL/6 mice and mice injected with PBS were used as control. Blood was collected at 1 day after injection for hematological analysis and serum biochemical tests. Major organs (heart, liver, spleen, lung and kidney) were collected for H&E staining.

### *In vivo* combinational therapeutic efficacy

All animal experiments were performed with the approval of the Ethics Committee of Affiliated Jinling Hospital, Medical School of Nanjing University. To build the bone metastatic breast cancer model, MDA-MB-231 cells (2 × 10^5^ cells in 20 μl PBS) were directly injected into the cavum medullare of 4–6 week-old female nude mice tibias. The tumor-bearing mice were divided into five groups, including control, m-CuS-PEG (material group), CDDP@m-CuS-PEG (chemotherapy group), m-CuS-PEG plus NIR laser (photothermal group), CDDP@m-CuS-PEG plus NIR laser (combinational therapy group). For the treatments, the experimental mice were intravenously administered with m-CuS-PEG or CDDP@m-CuS-PEG (4 mg/kg body weight) at 2, 4, and 6 d after the tumor volume reached ∼50 mm^3^. The mice of photothermal group and combined therapy group were irradiated with an 808 nm laser for 10 min. The treatments were performed once every 2 days. The tumor size and body weight of the mice were monitored every 2 days in the therapeutic duration. After the treatments, the tumors were excreted for photographed and H&E staining evaluation.

The tumor-bearing mice were placed in the scan chamber, and analyzed using a Biograph 3D micro-CT device (ZKKS-MCT-Sharp-I, Caskaisheng, China). After scanning, the 3D models were reconstructed and evaluated using the bone analysis software (ZKKS-MicroCT 4.1, Caskaisheng, China). In addition, bone mineral density (BMD) and trabecular number (Tb.N) were calculated automatically using the software.

## Results and discussion

### Preparation of the mesoporous nanoplatform CDDP@m-CuS-PEG

**FIGURE 2:**
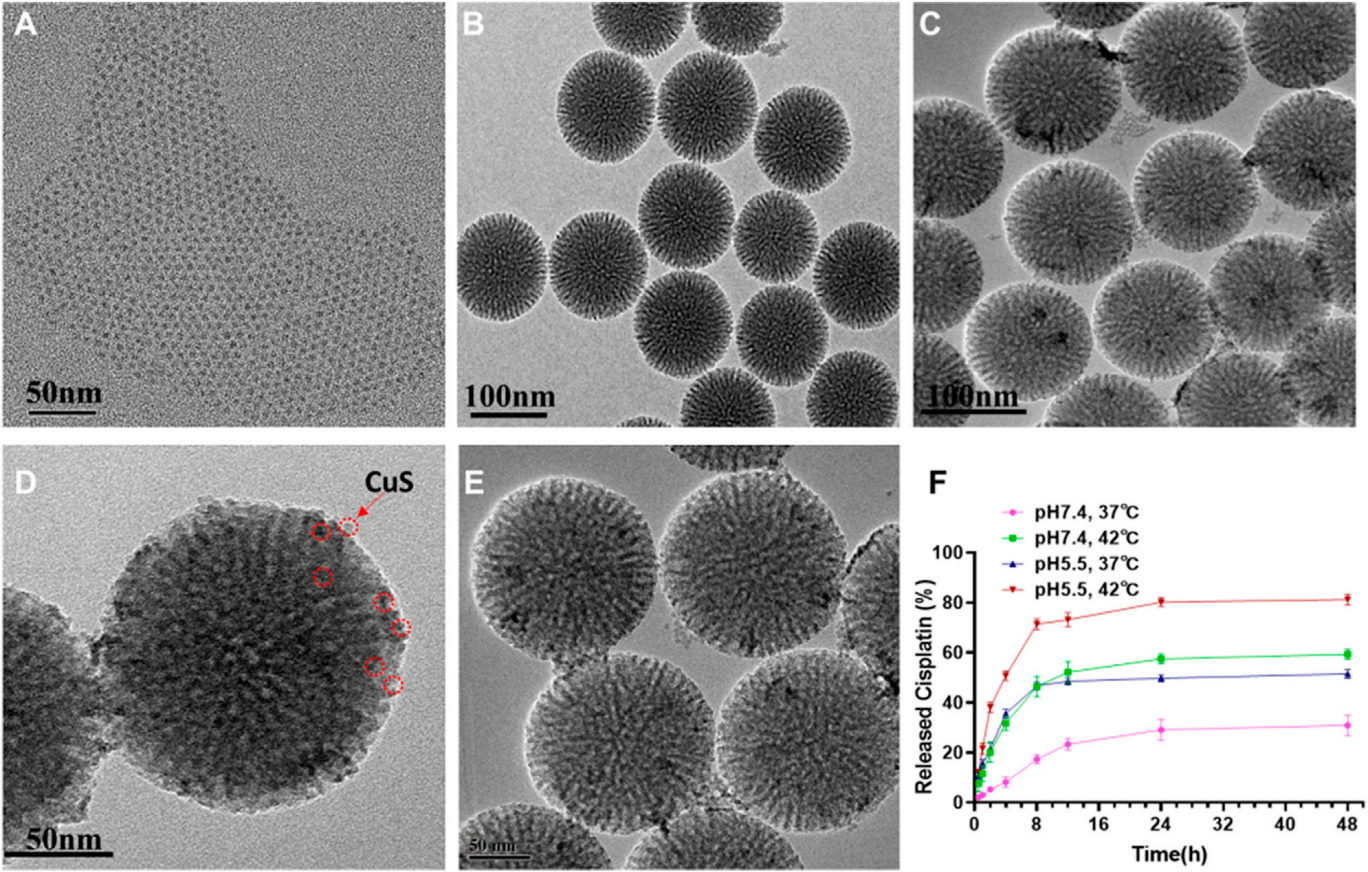
Characterization of CDDP@m-CuS-PEG NPs. **(A)** TEM image of CuS quantum dots; **(B)** TEM image of mSiO_2_-NH_2_ NPs; **(C,D)** TEM image of m-CuS-PEG NPs; **(E)** TEM image of CDDP@m-CuS-PEG; **(F)** Cisplatin release profile from CDDP@m-CuS-PEG at different pH conditions (pH 7.4 and pH 5.5) and temperature (37 or 42°C), respectively, *n* = 3.

To construct the functional nanoplatform, CuS nanodots were first prepared by a according to a previous report. (Xiao et al., 2013) The as-synthesized CuS nanodots showed an ultrasmall cluster-like morphology with a size of ∼5 nm, and the nanodots dispersed well with no apparent aggregation (Figure 2A, Supplementary Figure S1). mesoporous silica nanoparticles (mSiO_2_) with dendritic-like mesoporous channels were also successfully synthesized by bi-phase stratification approach. (Shen et al., 2014) Transmission electron microscope (TEM) image suggested that the average size of mSiO_2_ was ∼110 nm (Figure 2B). Upon the CuS nanodots were wrapped onto the mSiO_2_, black dots can be observed on the surface of the resulted m-CuS (Figure 2C). Enlarged TEM image indicated that the size and morphology of the black dots were in accordance with the CuS nanodots (Figure 2D). Moreover, it can be seen that the morphology and dispersity of m-CuS was not affected after the loading of cisplatin drugs and modification of PEG (Figure 2E). Dynamic light scattering (DLS) measurement was also conducted to assess the colloidal stability of prepared m-CuS-PEG. It is found that the size distribution of m-CuS-PEG shows no obvious change after 72 h incubation, indicating the high colloidal stability of the developed mesoporous nanoplatform. The porous structure of m-CuS-PEG is suitable to be loaded with small-molecular drugs. Here, the cisplatin was loaded into m-CuS-PEG and the loading content was calculated to be 28.5 mg/g.

Next, the drug release profile of cisplatin-loaded m-CuS-PEG (designated as CDDP@m-CuS-PEG) was further investigated. Under the normal physiological conditions, the cumulative releasing percentage of the loaded cisplatin was only 8% at (Figure 2F) the first 4 h, indicating that the undesirable leakage of the cisplatin can be blocked with the mesoporous carriers. Contrarily, the drug release rate was much faster under acidic pH condition (pH 5.5), with a high cumulative releasing percentage of 45% within 48 h. The pH-dependent drug release might be ascribed to the weakened electrostatic interaction between cisplatin and surface carboxyl groups of PEG. The fast drug release under acidic conditions is also beneficial for tumor treatment. In addition, the increase of temperature also accelerated the release of cisplatin. It was found that nearly 80% of loaded cisplatin could be released under pH 5.5 and 42°C. This may be attributed to the protonation of carboxyl groups in PEG, which weakens the interaction of drug molecules and the particles. (Gu et al., 2013; Park et al., 2019) Thus, the hybrid nanoplatform can realize more controlled drug release based on its pH and temperature dual-responsiveness. In view of the photothermal conversion ability of the CuS, NIR laser irradiation might be applicable to remotely accelerate the drug release *in vivo*.

### Photothermal effect of the mesoporous nanoplatform CDDP@m-CuS-PEG

**FIGURE 3:**
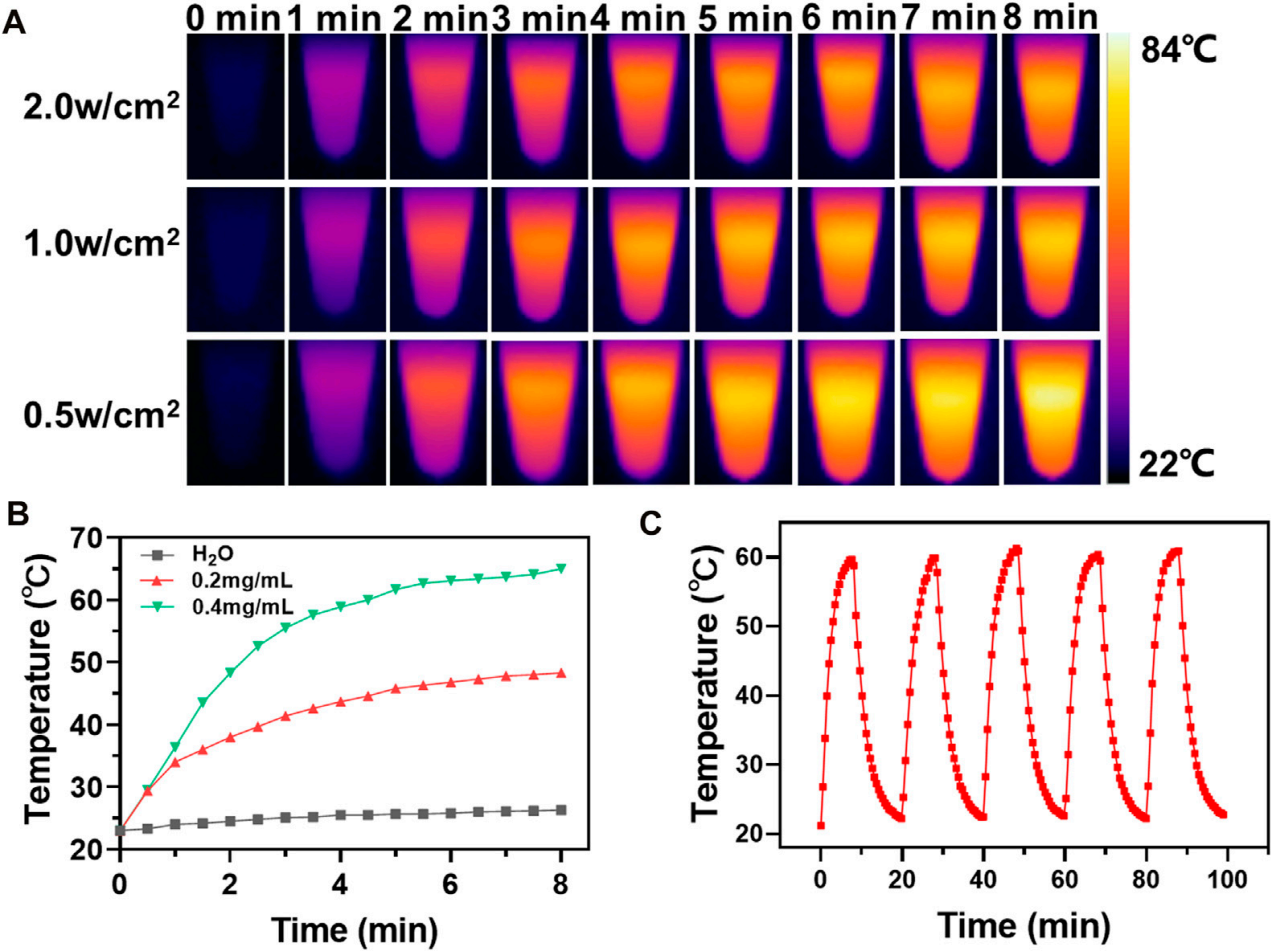
Photothermal effect of m-CuS-PEG NPs. **(A)** Photothermal performance of m-CuS-PEG (0.4 mg/ml) in aqueous solution under different NIR irradiation (808 nm, 0.5, 1.0, and 2.0 W/cm^2^) at different time points; **(B)** The temperature changes at indicated time *in vitro* with m-CuS-PEG at different concentrations. **(C)** Photothermal cycle stability curve.

As a representative photothermal agent, CuS is expected to impart favorable photothermal effect to the nanoplatform. So next, IR thermal imaging was employed to evaluate the photothermal effect of m-CuS-PEG. The aqueous dispersions of m-CuS-PEG were irradiated with the NIR laser at different power densities (0.5, 1, and 1.5 W/cm^2^) and the temperature of dispersions were recorded timely (Figure 3A). The rapid increase of temperature can be observed with the prolonged irradiation time. The thermal image of 400 μg/ml m-CuS-PEG solution showed a sharp color change from deep blue (low temperature) to bright yellow (high temperature). The increase range of temperature dependent on the power density of NIR laser. Furthermore, the m-CuS-PEG solution at different concentrations were irradiated with NIR laser for 8 min. The concentration-dependent temperature increase was also observed (Figure 3B). Specifically, the temperature can rise over 60°C after the irradiation. The results indicated that the prepared m-CuS-PEG can serve as an efficient photothermal agent, converting the NIR photonic energy into local hyperthermia. To evaluate the photothermal stability, m-CuS-PEG solution was subjected to NIR laser “on/off” cycles. As shown in Figure 3C, the elevation range of temperature has no obvious change after five cycles, which corroborated the high photostability of m-CuS-PEG under NIR laser irradiation. Besides, the photothermal conversion efficiency of m-CuS-PEG was calculated to be 29.3%. Taken together, the decoration of CuS onto mSiO_2_ can permit the good photothermal effects of m-CuS-PEG for therapeutic application.

### *In vitro* therapeutic efficacy of CDDP@m-CuS-PEG

**FIGURE 4:**
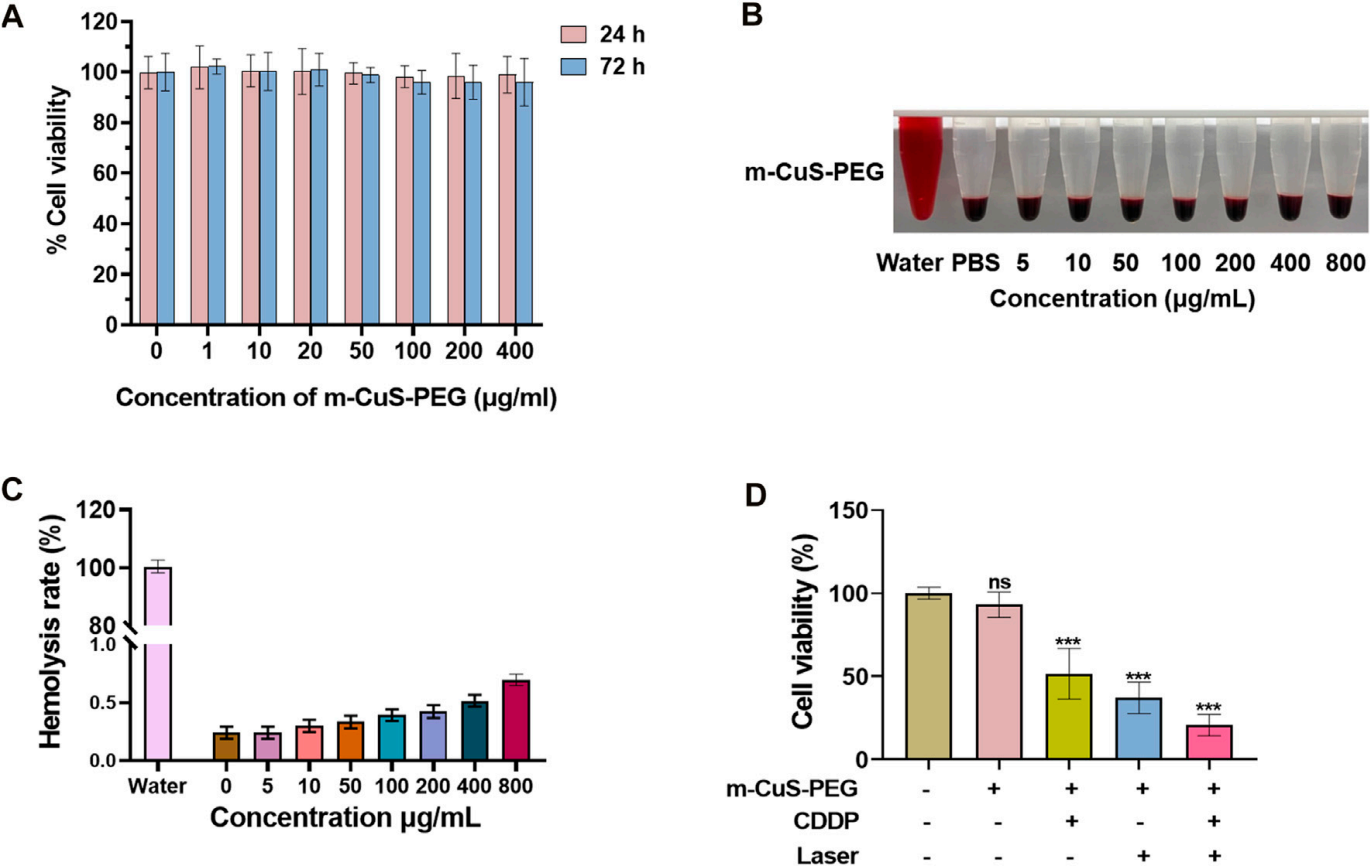
*In vitro* photothermal killing effect of CDDP@m-CuS-PEG on tumor cells. **(A)** Cell viability assay of MDA-MB-231 cells incubated with m-CuS-PEG at different concentrations (0, 1, 10, 20, 50, 100, 200 and 400 μg/ml) under NIR laser irradiation for 10 min every 12 h. **(B)** Cell viability assay of MDA-MB-231 cells incubated with m-CuS-PEG, free CDDP, m-CuS-PEG + Laser and PDD@m-CuS-PEG + Laser (100 μg/ml); **(C,D)** Hemolytic properties of m-CuS-PEG at different concentrations. All sample sizes are 3.

The dual-responsive drug release and photothermal effect of CDDP@m-CuS-PEG are expected to kill cancer cells effectively. Thus, cell experiments were performed using human MDA-MB-231 breast cancer cells. Firstly, the cytotoxicity of drug-free m-CuS-PEG was evaluated by the standard CCK-8 assay. MDA-MB-231 cells were incubated with m-CuS-PEG dispersions at varied concentrations (1, 10, 20, 50, 100, 200, and 400 μg/ml). After 24 h incubation, the relative cell viability was all higher than 90% (Figure 4A). The relative viability of treated cells has no abnormal decrease even incubated for 72 h. Moreover, the cytotoxicity of m-CuS-PEG towards mammary epithelial MCF-10A cells was investigated. It can be observed that m-CuS-PEG exhibited no damage to MCF-10A cells even at high concentrations (Supplementary Figure S3). The results of CCK-8 assay confirmed the excellent biocompatibility of m-CuS-PEG. Additionally, the hemocompatibility of m-CuS-PEG was preliminarily assessed by the hemolytic assay. Red blood cells were exposed to m-CuS-PEG and the absorbance at 540 nm of the collected supernatants was measured to determine the hemolytic percentage. As presented in Figure 4C, the supernatants of all samples were completely transparent, which corroborated that the structural integrity of red blood cells maintained well regardless of the concentration of m-CuS-PEG. Moreover, the quantitative result of hemolytic percentage showed that no hemolytic effects occurred even at a high concentration of 800 μg/ml (lower than 1%) (Figure 4D). Therefore, the results demonstrated the prominent biocompatibility of m-CuS-PEG.

Next, the *in vitro* therapeutic efficacy of CDDP@m-CuS-PEG was evaluated. The MDA-MB-231 cancer cells treated with different samples and operation, including control, CDDP@m-CuS-PEG, m-CuS-PEG + laser, and CDDP@m-CuS-PEG + laser. After the corresponding treatments, the relative cell viability of CDDP@m-CuS-PEG group and m-CuS-PEG + laser group were both decreased obviously (Figure 4B). More than 50% of cancer cells were killed by the chemotherapeutic and photothermal effect, respectively. More importantly, the cell viability of the combinational group (CDDP@m-CuS-PEG + laser) was only ∼19.5%. It is also noted that the CDDP@m-CuS-PEG had lower therapeutic efficacy than the combination treatment at the same concentration of 100 μg/ml (Supplementary Figure S4). Collectively, the results demonstrated that the combined therapy could realize superior efficacy by virtue of the photothermal effects and responsive local drug release.

### *In vivo* biosafety evaluation of m-CuS-PEG

**FIGURE 5:**
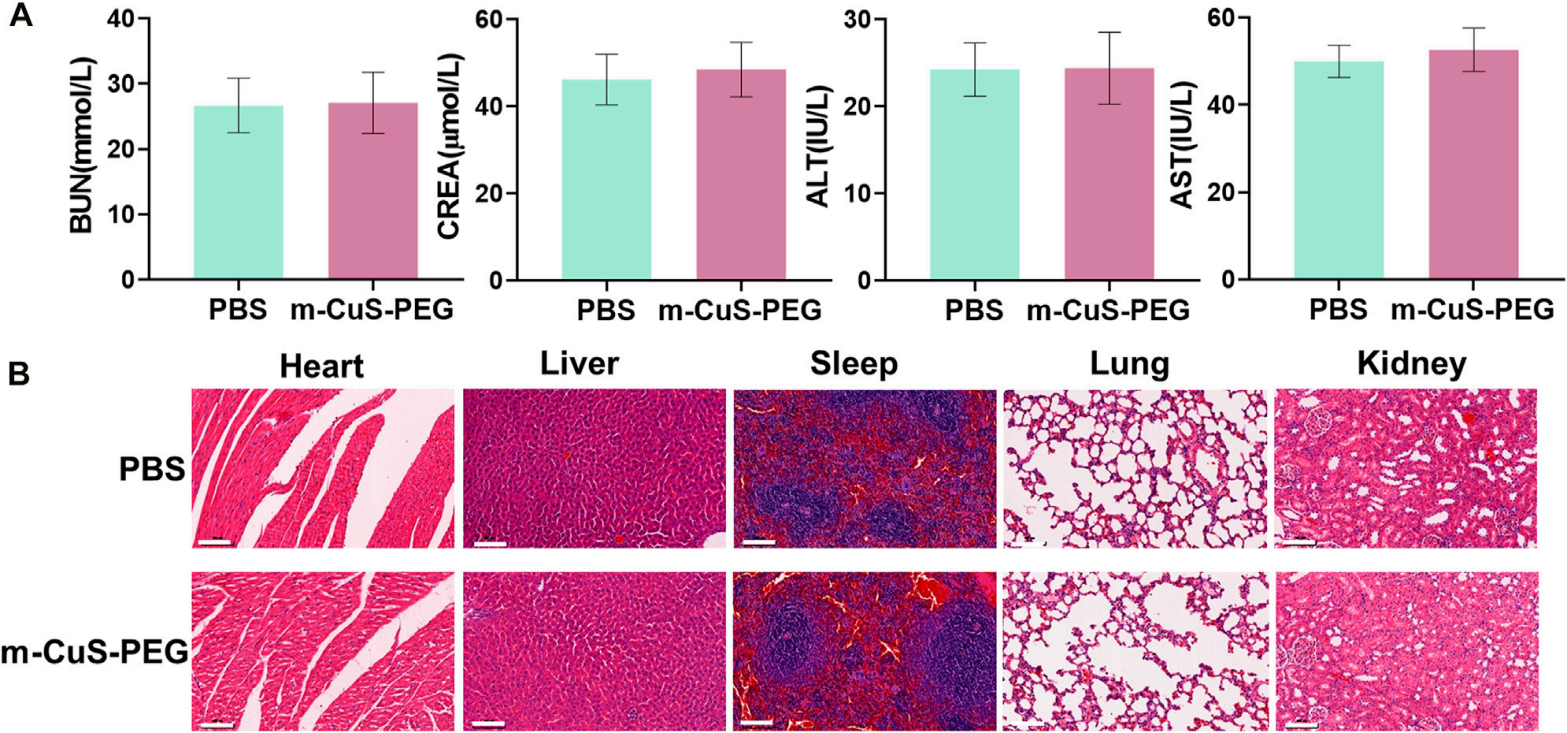
Biosafety of m-CuS-PEG. **(A)** Liver function (ALT, AST) and kidney (BUN, CREA) function of m-CuS-PEG. sample size: *n* = 3; **(B)** H&E staining images of mice major organs after PBS or m-CuS-PEG treatment. Scale bars = 100 μm for H&E.

In addition, the potential *in vivo* toxicity of the nanoplatform m-CuS-PEG was investigated in terms of blood chemistry and histological evaluation. The dispersion of m-CuS-PEG was intravenously injected into healthy Balb/c mice at a dose of 20 mg/kg. Then, the blood of the experimental mice was collected from the eyeballs after 1 day injection. The samples were sent for serum biochemistry examination. As shown in Figure 5A, the serum parameters of m-CuS-PEG treated mice exhibited no abnormal values as compared with the control group, involving the liver function indexes of alanine aminotransferase (ALT) and aspartate aminotransferase (AST), the kidney function markers of blood urea nitrogen (BUN) and creatinine (CREA). Moreover, the major organs (heart, liver, spleen, lung, and kidney) of the treated mice were excreted, sliced, and stained with hematoxylin and eosin (H&E) for histological evaluation after 2 weeks of intravenous injection of m-CuS-PEG. The results also indicated that no perceptible tissue damage or inflammatory lesion was observed in all tissue samples, which is similar with the control group (Figure 5B). Thus, the above results demonstrated that the developed m-CuS-PEG has negligible toxicity *in vivo*, permitting its safe applications for therapy. Despite the favorable biocompatibility of m-CuS-PEG, its biodegradation *in vivo* is unsatisfactory, which might impede the clinical translation of silica-based biomaterials. To address this issue, the long-term toxicity and biodistribution of the mesoporous nanoplatform could be investigated in a more rigorous manner.

### *In vivo* antitumor efficacy of CDDP@m-CuS-PEG on metastasized breast cancer therapy

Given the combined therapeutic effects and biocompatibility of the nanoplatform, the *in vivo* antitumor efficacy was further investigated by using a metastasized breast tumor model. MDA-MB-231 cancer cells were injected into the cavum medullare of nude mice tibias for establishing bone metastasized tumor model. The tumor accumulation of m-CuS-PEG was first explored by fluorescent imaging. Indocyanine green (ICG)-labeled m-CuS-PEG was injected into the tumor-bearing mice and the fluorescent images at different time points were captured. The m-CuS without PEG modification was also labeled with ICG and set as control for comparison. After 1 h injection, fluorescent signals can be detected in all two groups of m-CuS-PEG and m-CuS (Supplementary Figure S5A). Notably, the fluorescent intensity of m-CuS-PEG group was much stronger than that of the unmodified m-CuS within a long period post-injection. At 36 h, the major tissues were excreted for measurements. It was found that markedly enhanced fluorescence emerged in the tumor and bone sites for m-CuS-PEG group. In comparison, the brightness of fluorescence signals significantly decreased for the m-CuS-PEG (Supplementary Figure S5B). The same trends were also corroborated in the results of semi-quantitative fluorescent intensity values (Supplementary Figure S5C), suggesting that the modification of carboxylated PEG onto m-CuS can improve its specific accumulation in tumor sites. The enhanced tumor accumulation of m-CuS-PEG may be ascribed to its improved pharmacokinetics based on the stealth character of PEG. It is also noted that the accumulation amount of m-CuS-PEG in liver was much lower than the bare m-CuS, which is primarily due to that PEG can reduce the protein adsorption and mitigate the opsonization. In addition, the carboxyl-terminated surface may also endows the bone targeting ability with the nanoplatform because of high affinity of carboxyl groups to hydroxyapatite and bone fragments according to previous study. (Wang et al., 2007; Yamashita et al., 2017; Yan et al., 2019) Therefore, the developed nanoplatform m-CuS-PEG is anticipated to effectively accumulate at the tumor and metastasized bone lesions to exert its synergistic therapeutic efficacy.

**FIGURE 6:**
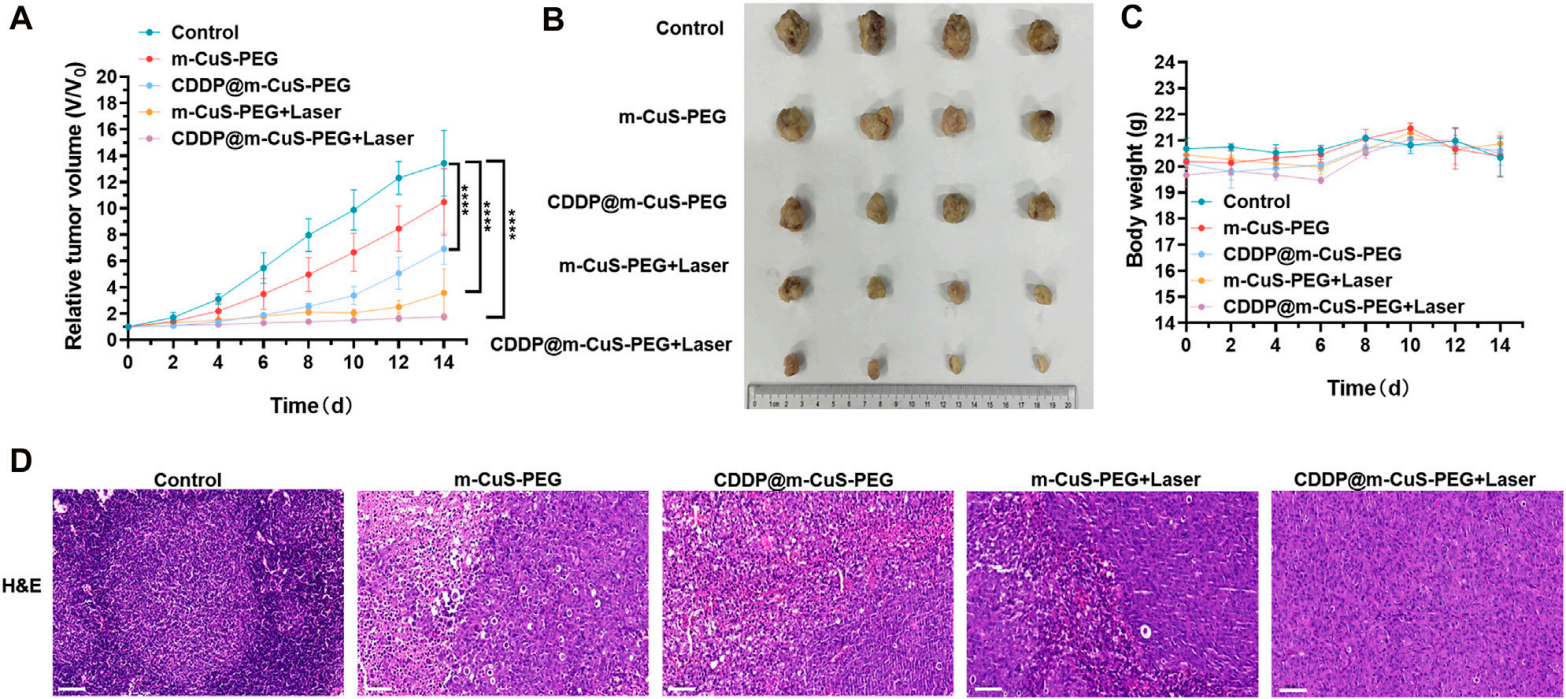
*In vivo* antitumor efficacy of CDDP@m-CuS-PEG on metastasized breast cancer therapy. **(A)** Tumor volumes relative to the start point in each group collected on day 14, sample size: *n* = 4. **(B)** Images of tumor-bearing mice on days 2, 4, 6, 8, 10, 12 and 14. **(C)** Weight changes curve within 14-day treatments of each group, sample size: *n* = 4; **(D)** H&E staining of tumor sections in each group. Scale bars = 100 μm for H&E.

To evaluate the *in vivo* tumor therapeutic efficacy, the tumor-bearing mice were randomly divided into different groups: control, m-CuS-PEG, CDDP@m-CuS-PEG, m-CuS-PEG + laser, and CDDP@m-CuS-PEG + laser. The tumor volume and body weight of the mice were measured after the corresponding treatments. As presented in Figure 6A, in the control group, the tumor volume significantly increased after 2 weeks. Specifically, the average tumor volume increased by over 14 times compared with the original tumor volume at the beginning of treatment for the control group, and over 10.5 times for the m-CuS-PEG group, indicating that the nanoplatform alone has negligible tumor inhibition efficacy *in vivo*. In the case of CDDP@m-CuS-PEG-injected group, the average tumor volume slowly increased to 600% compared with the initial tumor volumes. Moreover, the photothermal group of m-CuS-PEG + NIR laser showed a higher inhibition rate of tumor growth than the mere chemotherapy group of CDDP@m-CuS-PEG. By contrast, the tumor size also maintained unchanged compared with the original size under the combined treatment of CDDP@m-CuS-PEG plus NIR laser irradiation. The photographs of excreted tumors also confirmed the efficient tumor growth inhibition of the group of synergistic therapy (Figure 6B). The body weight of the experimental mice showed no abnormal change during the monitoring period of treatment (Figure 6C), indicating the implemented treatments were well-tolerant without adverse effects. Furthermore, H&E staining of tumor slices was also performed to visualize the cancer cells in tumor. Similarly, suppressed cell proliferation was presented in the group of CDDP@m-CuS-PEG plus NIR laser irradiation compared with other groups (Figure 6D). The results demonstrate that the photothermal-chemotherapy enabled by CDDP@m-CuS-PEG can efficiently eradicate the tumors *in vivo*, and the combined therapeutic efficacy was remarkably superior to the antitumor effect of the monotherapy.

Bone metastasized tumor is inclined to induce osteoclastic bone resorption, leading to a series of complications, such as bone pain and bone loss, which severely threats the life quality of patients and increases the mortality. (Gartrell and Saad 2014; Wang et al., 2017) Hence, it is also critical to inhibit osteoclastic bone resorption around tumor sites in addition to the depression of tumor growth. In view of this, the morphological change of bone of tumor-bearing mice was evaluated by CT imaging. The 3D reconstructed images of tibias indicated that the mice treated with combined photothermal and chemotherapy exhibited minimal bone deconstruction (Supplementary Figures S6A–C). The 3D architecture parameters, including bone mineral density (BMD) and trabecular numbers (Tb), were also summarized and compared. Both the BMD and Tb numbers of the tibias of mice treated with CDDP@m-CuS-PEG + laser were larger than that of other groups (Supplementary Figures S6D,E). Moreover, the spontaneous lifting of the affected limb of the tumor-bearing mice was also recorded during the treatments. As expected, the number of flinches for the combinational therapy group was significantly reduced in comparison to the control group and other treatment groups (Supplementary Figure S7), indicating that the treatment of CDDP@m-CuS-PEG + laser can effectively attenuate the cancer pain to some extent. The presented data revealed that m-CuS-PEG mediated synergistic photothermal-chemotherapy not only eradicated the metastasized tumors but also attenuated the tumor-induced osteoclastic destruction.

## Conclusion

In this work, we developed a mesoporous naonplatform m-CuS-PEG, in which ultrasmall CuS nanodots were uniformly decorated on the surface of dendritic mSiO_2_ nanoparticles and carboxyl-terminated PEG was immobilized on the outmost layer. The mesopores of mSiO_2_ can be utilized to load chemotherapeutic drugs cisplatin, and the drug release manner exhibited typical pH/thermal dual-responsiveness. The developed m-CuS-PEG had remarkable photothermal effects upon NIR laser irradiation. The temperature of the dispersions at a concentration of 400 μg/ml can rise over 60°C after 8 min irradiation. Moreover, the high stability of m-CuS-PEG under repeated NIR laser irradiation was demonstrated. Thus, the photothermal effect and responsive chemotherapeutic release of the drug-loaded nanoplatform (CDDP@m-CuS-PEG) resulted in highly efficient killing of MDA-MB-231 cancer cells based on the synergistic therapy. *In vitro* and *in vivo* evaluations also corroborated the outstanding biocompatibility of m-CuS-PEG. In addition, the synergistic chemo-photothermal therapy significantly eradicated the bone metastasized tumor *in vivo*, compared with the single-modality photothermal therapy or chemotherapy. Importantly, 3D reconstructed CT images indicated that the metastasized tumor-induced bone resorption was profoundly suppressed under the combined treatment, which can be attributed to the enhanced accumulation of m-CuS-PEG in the tumor lesions around the bone. Therefore, this study demonstrates that the m-CuS-PEG with the combination of photothermal therapy and responsive drug delivery capability holds great potential an efficient nanoagent for the effective therapy of breast cancer bone metastases.

## Data Availability

The raw data supporting the conclusion of this article will be made available by the authors, without undue reservation.
