# Supplementary material for: Mesoporous nanoplatform integrating photothermal effect and enhanced drug delivery to treat breast cancer bone metastasis
Source: Front Chem. 2022 Nov 30;10:1088823. doi: 10.3389/fchem.2022.1088823 (PMC9749821; doi:10.3389/fchem.2022.1088823)
Supplement: Supplementary file 1 [file DataSheet1.docx]

Supplementary Material

# 1.Supplementary Experimental Section

**1.1 Materials and characterization.**

***1.1.1 Materials.***

Cetyltrimethylammonium bromide (CTAB) and tetraethylorthosilicate (TEOS) was purchased from Sigma-Aldrich (St. Louis, MO, USA). CuCl_2_•2H_2_O, Na_2_S•9H_2_O, NaOH, and anhydrous ethanol were obtained from Sinopharm Chemical Reagent Co. (Beijing, China). (3-aminopropyl)triethoxysilane (APTES), polyvinylpyrrolidone (PVP), sodium citrate, and triethanolamine (TEA) were purchased from Aladdin Chemistry Co., Ltd (Shanghai). 1-(3-dimethylaminopropyl)-3-ethylcarbodiimide hydrochloride (EDC•HCl) and N′-hydroxysuccinamide (NHS) were purchased from Admas Pharmaceuticals, Inc. Carboxyl-terminated PEG was commercially available from Ponsure biological Co. Ltd (Shanghai). All chemicals were of analytic grade and used without further purification. MDA-MB-231 breast cells were purchased from ATCC (Manassas, VA, USA).

***1.1.2 Characterization.***

Transmission electron microscopy (TEM) measurements were carried out on a JEM 2100F microscope (Japan) operated at 200 kV.

1. **Supplementary Figures Section**


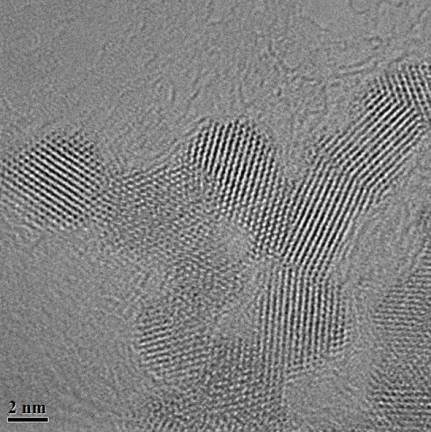


**Figure S1.** TEM image of CuS quantum dots.


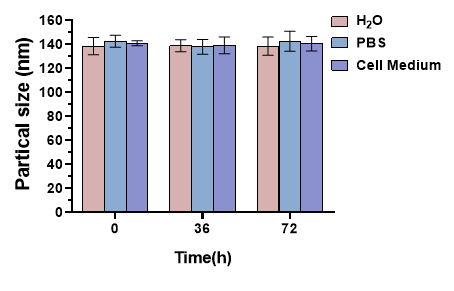


**Figure S2.** The size distribution of m-CuS-PEG dispersed in different media at different time points.


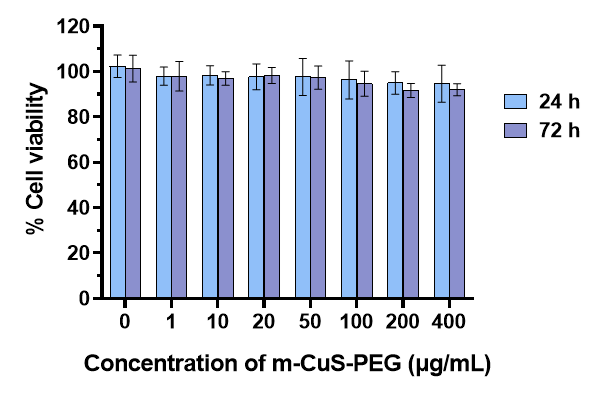


**Figure S3.** Cell viability of MCF-10A cells incubated with different concentration m-CuS-PEG for 24 h.


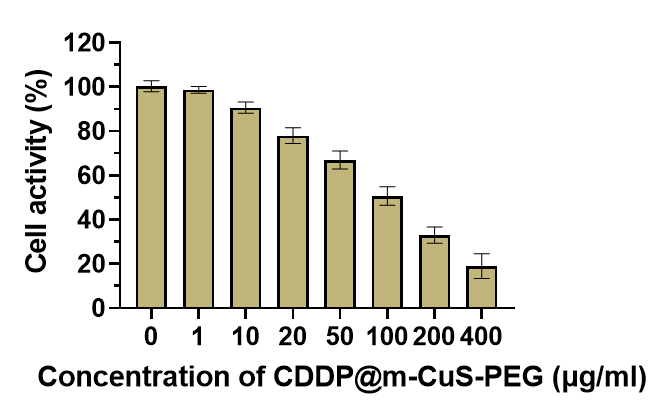


**Figure S4.** Cell viability of MDA-MB-231 cells incubated with different concentration CDDP@m-CuS-PEG for 24 h.


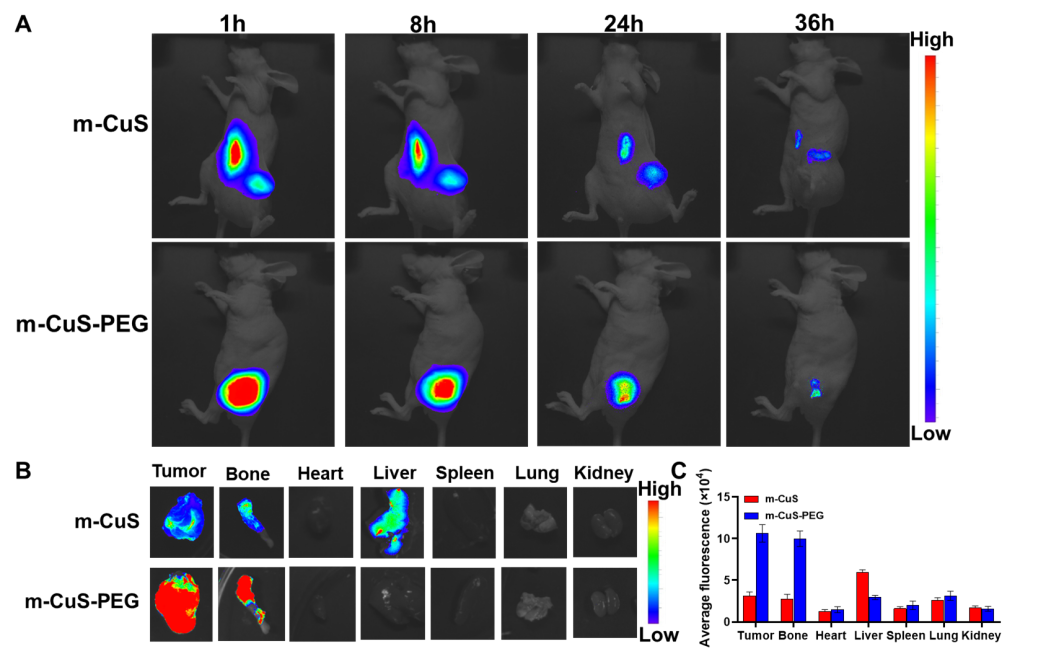


**Figure S5.** (A) *In vivo* fluorescent imaging of tumor-bearing mice after intravenous injection of m-CuS@ICG and m-CuS-PEG@ICG at indicated time points; (B) Ex vivo fluorescence signal of excised tumors and organs at 36 h after injection; (C) Quantitive analysis of signal intensity in each organ of (B).


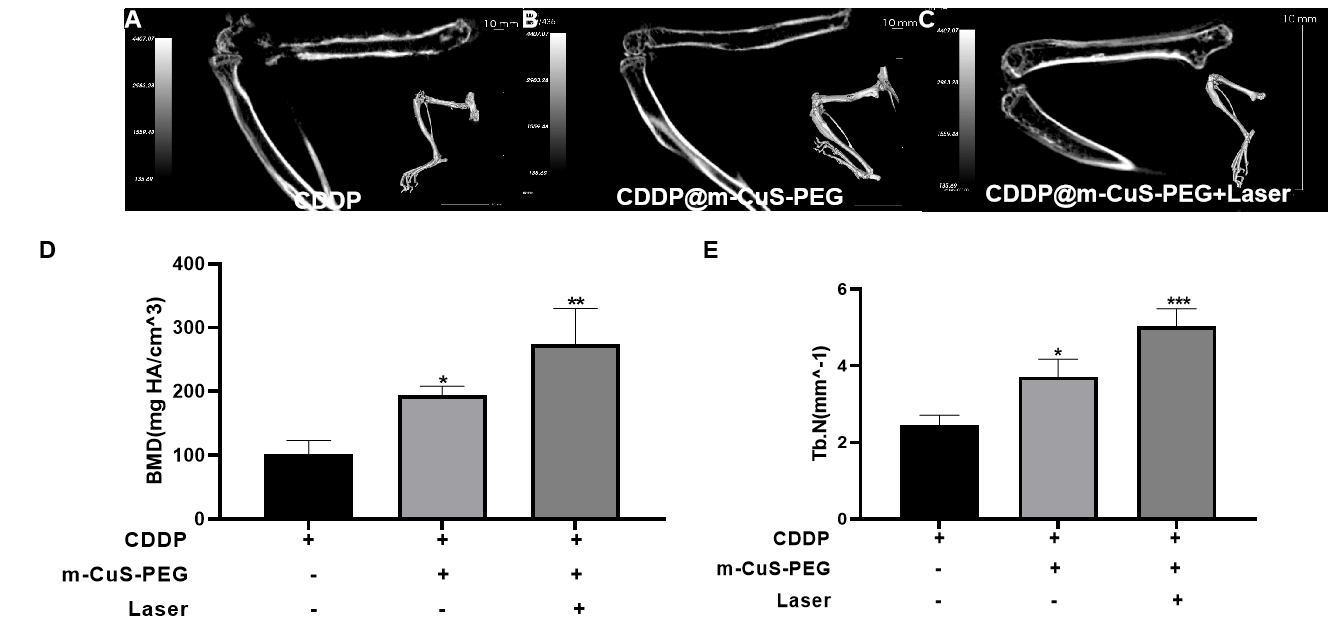


**Figure S6.** Evaluation of bone destruction. (A) Micro-CT 3D reconstruction image, the 2D transverse sections of tibia, and the longitudinal sections. (B) BMD, (C) Tb.N. Tumor bearing mice were pretreated with CDDP, CDDP@m-CuS-PEG NPs, or CDDP@m-CuS-PEG+Laser.


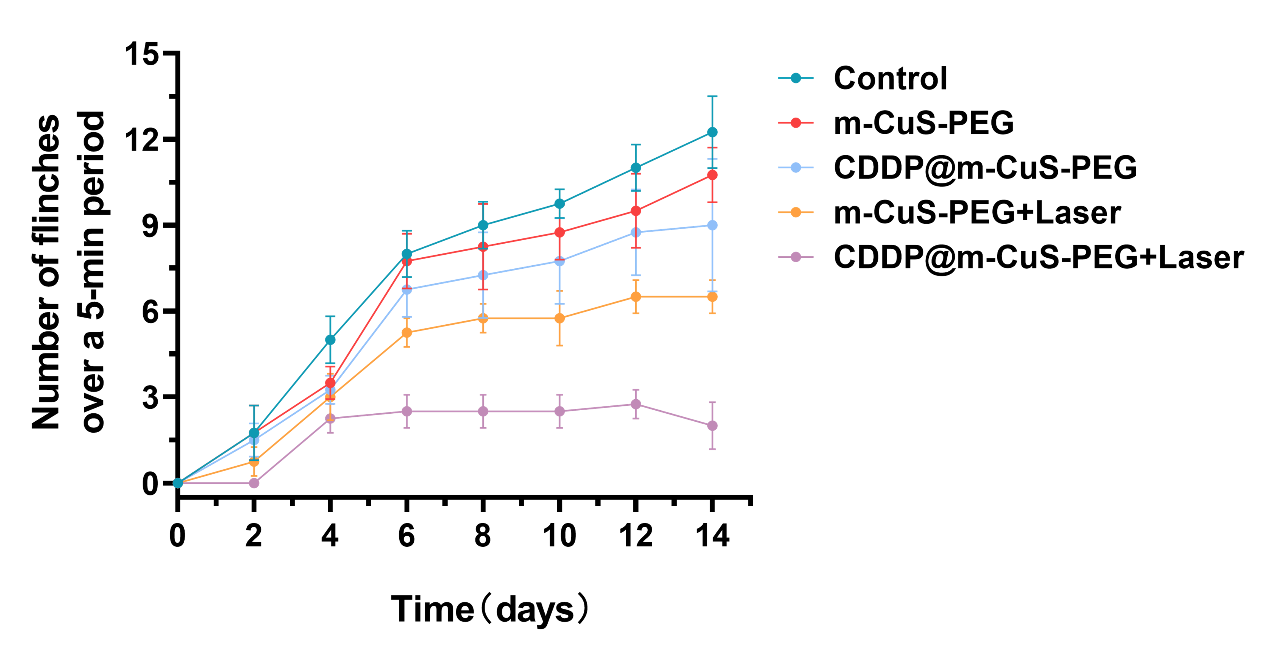
 **Figure S7.** The number of leg lifts at metastatic tumor site in 5 min.
